# Antibody-Dependent Enhancement Activity of a Plant-Made Vaccine against West Nile Virus

**DOI:** 10.3390/vaccines11020197

**Published:** 2023-01-17

**Authors:** Haiyan Sun, Dhiraj Acharya, Amber M. Paul, Huafang Lai, Junyun He, Fengwei Bai, Qiang Chen

**Affiliations:** 1 The Biodesign Institute and School of Life Sciences, Arizona State University, Tempe, AZ 85287, USA; 2 Department of Cell and Molecular Biology, University of Southern Mississippi, Hattiesburg, MS 39406, USA

**Keywords:** West Nile virus (WNV), vaccine, antibody-dependent enhancement (ADE), virus-like particle (VLP), domain III (DIII), dengue virus (DENV), Zika virus (ZIKV), plant-produced vaccine

## Abstract

West Nile virus (WNV) causes annual outbreaks globally and is the leading cause of mosquito-borne disease in Unite States. In the absence of licensed therapeutics, there is an urgent need to develop effective and safe human vaccines against WNV. One of the major safety concerns for WNV vaccine development is the risk of increasing infection by related flaviviruses in vaccinated subjects via antibody-dependent enhancement of infection (ADE). Herein, we report the development of a plant-based vaccine candidate that provides protective immunity against a lethal WNV challenge mice, while minimizes the risk of ADE for infection by Zika (ZIKV) and dengue (DENV) virus. Specifically, a plant-produced virus-like particle (VLP) that displays the WNV Envelope protein domain III (wDIII) elicited both high neutralizing antibody titers and antigen-specific cellular immune responses in mice. Passive transfer of serum from VLP-vaccinated mice protected recipient mice from a lethal challenge of WNV infection. Notably, VLP-induced antibodies did not enhance the infection of Fc gamma receptor-expressing K562 cells by ZIKV or DENV through ADE. Thus, a plant-made wDIII-displaying VLP presents a promising WNV vaccine candidate that induces protective immunity and minimizes the concern of inducing ADE-prone antibodies to predispose vaccinees to severe infection by DENV or ZIKV.

## 1. Introduction

West Nile virus (WNV) infection in humans can cause severe neuroinvasive diseases including encephalitis, meningitis, and even death [1]. The elderly and individuals who are immunocompromised or those carry certain genetic factors are at a higher risk of developing life-threatening and fatal neurological diseases [2,3]. WNV used to be an old-world virus, but it has spread to the rest of the world, causing frequent outbreaks with more patients exhibiting neuroinvasive complications in recent years [4]. However, there is still no licensed WNV vaccine for human use.

WNV is a member of the genus *Flavivirus* in the family *Flaviviridae,* and is genetically closely related to dengue virus (DENV), Zika virus (ZIKV), tick-borne encephalitis virus (TBEV), and yellow fever virus (YFV) [1]. WNV envelope glycoprotein (wE) shares the three-domain structures (wDI, wDII, and wDIII) with other flaviviruses [5] and mediates viral assembly, attachment to cellular receptors, and the subsequent membrane fusion for viral entry [6]. wE is also a major target for the host immune response and the majority of type-specific neutralizing and protective epitopes are localized in wDIII [7]. Since neutralizing antibody responses have been shown to correlate with protection for licensed vaccines against YFV and TBEV, and for vaccine candidates against WNV [8,9], wDIII is considered a favorable WNV vaccine candidate due to the presence of multiple neutralizing epitopes in this domain.

The high degree of genetic similarity between WNV and related flaviviruses such as ZIKV and DENV presents challenges for vaccine safety because of the phenomenon called antibody-dependent enhancement of infection (ADE). ADE has been shown to be clinically relevant to DENV infection [10]. Specifically, some antibodies elicited during a primary infection by a specific DENV serotype are non-protective against a different DENV serotype in a secondary infection, but instead, can enhance its infection in Fc gamma receptor (FcγR)-expressing cells, leading to a potentially lethal shock syndrome through ADE [11]. Therefore, WNV vaccines based on conserved epitopes among related flaviviruses would have the risk of evoking cross-reactive antibodies that augment entry and replication of DENV and ZIKV in FcγR-bearing cells and lead to severe DENV or ZIKV infection in vaccinated subjects via ADE [11]. Indeed, mutual enhancement between WNV and ZIKV infections has been already observed [12]. Thus, human WNV vaccines should be not only potent but also safe with a minimal risk of inducing ADE.

We previously reported our effort in developing a WNV vaccine candidate in plants using a chimeric hepatitis B core antigen (HBcAg) virus-like particle (VLP) that displays wDIII on its surface (HBcAg-wDIII VLP) [13]. We demonstrated that HBcAg-wDIII VLP was expressed at high levels rapidly in *Nicotiana benthamiana* plants and immunization of plant-produced HBcAg-wDIII VLP evoked wDIII-specific antibody response in mice. Here, we report a follow-up study of the efficacy and safety of HBcAg-wDIII VLP as a promising vaccine against WNV. The neutralizing potency of wDIII-specific antibodies, the antigen-specific cellular immune responses, and the protectivity of HBcAg-wDIII VLP immunization in mice against a lethal challenge are investigated. Furthermore, the risk of ADE by this vaccine candidate in enhancing ZIKV and DENV infection is evaluated to address the potential safety issue.

## 2. Material and Methods 

### 2.1. HBcAg-wDIII VLP Production in Plants

HBcAg-wDIII VLPs were produced in *N. benthamiana* leaves as described previously [14,15,16]. Leaves were harvested 7 days post agroinfiltration (dpi) and HBcAg-wDIII VLPs were extracted and purified with sucrose gradient centrifugation as previously described [13].

### 2.2. Mouse Immunization

Five-week-old female BALB/c mice were used for immunization. Mice were divided into 2 groups (*n* = 6 per group), with group 1 receiving 100 μL PBS saline buffer (PBS) with adjuvant aluminum hydroxide gel (alum, Sigma, Burlington, MA, USA) as a mock immunization control, and group 2 receiving 100 μL material containing 25 μg of HBcAg-wDIII VLPs in PBS with alum as adjuvant per dosage. Mice were primed on day 0 with subcutaneous injection and were boosted three times on days 21, 42, and 63 with the same dosage and immune protocol as in the prime immunization. Retro-orbital (r.o.) blood samples were collected on day 0 before the immunization (pre-immune sample) and on days 14 (week 2), 35 (week 5), and 56 (week 8) after the 1st immunization. Final blood samples were collected on day 77 (week 11) after mice were humanely euthanized. The spleens were aseptically removed after euthanization for in vitro splenocyte cultures.

### 2.3. Antibody Neutralization Assay

The neutralizing potency of wDIII-specific antibodies was measured with a plaque reduction neutralization test (PRNT) assay as we previously reported [17,18,19]. Neutralizing antibody titers were expressed as the reciprocal of the highest dilution of serum that neutralized ≥50% of WNV. PRNT assay details are provided in the Supplementary Material.

### 2.4. Cytokine Production in Splenocyte Culture

Spleens were isolated from immunized mice and mechanically dissociated to establish single-cell splenocyte cultures. Cytokine production in splenocyte cultures was then determined as described previously [20]. Method details are provided in the Supplementary Material.

### 2.5. ADE Assay

Total IgG was isolated from pooled sera collected from vaccinated mice at week 11 as described previously, and ADE assay was then performed following our published protocol [17,21,22], with details provided in the Supplementary Material.

### 2.6. Mouse Protection Experiment

Passive serum transfer followed by WNV challenge was performed to assess the in vivo potency of the HBcAg-wDIII VLP vaccine. Serum isolated from PBS or HBcAg-wDIII VLP-immunized mice at week 11 was heat-inactivated for 30 min at 56 °C. Five-week-old female BALB/c mice were anesthetized with 25% isoflurane and passively administered serum via retro-orbital injection. Specifically, mice were divided into 3 groups (*n* = 10 per group), with Group 1 receiving 50 µL of serum from PBS mock-immunized mice, and Group 2 receiving 50 µL of serum from HBcAg-wDIII VLP-immunized mice, respectively. Mice in Group 3 received 10 µg of a protective monoclonal antibody (mAb) E16 [23] as a positive control. One hour after receiving serum or mAb, mice were challenged with 10^2^ plaque-forming units (PFU) of WNV (CT2741, provided by Dr. John F. Anderson at the Connecticut Agricultural Experiment Station) in 1% gelatin via intraperitoneal (i.p.) inoculation. The survival of mice after viral challenge was monitored for 25 days. Data used for generating the survival curves are from two independent experiments. 

### 2.7. Statistical Analyses

Data were statistically analyzed using GraphPad Prism software version 8.4 (GraphPad, CA, USA). Student’s *t*-test was used to compare serum neutralization potencies between different mouse groups. One-way ANOVA and two-way ANOVA were used to compare concentrations of cytokines between mouse groups and between samples collected at various time points. Mouse survival from at least two independent WNV challenge experiments (*n* = 10) were analyzed by a Log-rank (Mantel-Cox) analysis. A *p* value of <0.05 was used to indicate statistically significant difference.

## 3. Results

### 3.1. HBcAg-wDIII VLPs Induced Potent Neutralizing Antibody Responses against WNV

BALB/c mice received either four doses of HBcAg-wDIII VLPs with alum as an adjuvant or saline (PBS + alum, mock immunization control) over an 11-week time period with blood collected before immunization, and at 2 weeks, 5 weeks, and 8 weeks after the prime immunization. We previously reported that HBcAg-wDIII VLPs elicited high titers of wDIII-IgG responses (log titers > 3.4 and 4.3 for week 5 and 8, respectively) [13]. We also reported that HBcAg-wDIII VLPs with alum as the adjuvant induced both IgG1 and IgG2 subtype responses with higher titers of IgG1 detected than IgG2a [13].

**Figure 1:**
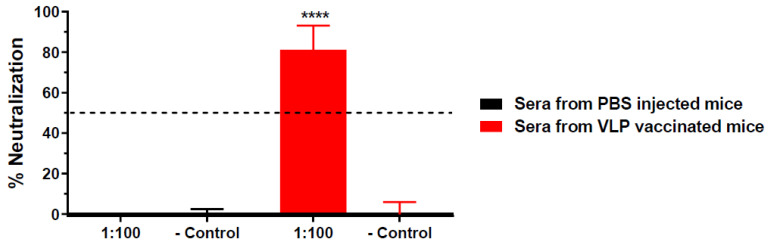
**Neutralization of WNV by serum from HBcAg-wDIII VLP immunized mice.** The neutralization potency against WNV of pooled sera from mice that were immunized with HBcAg-wDIII VLP (VLP) or mock-immunized with PBS collected at 5 week post-immunization was measured by a PRNT assay. Sera were diluted 100-fold and incubated with 10^2^ PFU of WNV prior to Vero cell infection. Mean % neutralization and SD from two independent experiments with technical triplicates for each sample are presented. − Control: PBS buffer in the PRNT assay. **** indicates *p* values < 0.0001 of HBcAg-wDIII VLP-immunized sera compared to sera from PBS-inoculated mice.

Neutralization potency of HBcAg-wDIII VLP-induced antibodies was assessed by a PRNT assay with sera collected 2 and 8 weeks after the first boost injection (using week 5 and 11 sera). As shown in Figure 1, week 5 sera from HBcAg-wDIII VLP-immunized mice exhibited high neutralization potency against WNV; meanwhile, such activity was not observed for sera of PBS-injected mice (*p* < 0.0001 comparing anti-HBcAg-wDIII VLP sera with PBS sera). Specifically, anti-HBcAg-wDIII VLP sera diluted 100-fold reduced WNV infection by 81% (Figure 1). Furthermore, 100-fold-diluted week 11 sera from HBcAg-wDIII VLP-immunized mice reduced WNV infection by 100% (Figure S1, *p* < 0.0001 compared to week 11 sera from PBS-injected mice, *p* = 0.0315 compared to week 5 sera of the same mouse group). Neutralization titers that correlate with protection in humans and animal models have been established for several flaviviruses including WNV, with neutralization titers >10 being the threshold for providing protective immunity [20,22,24,25,26,27]. Our results indicate that HBcAg-wDIII VLPs elicited an antibody response with a neutralization titer of >100 as early as week 5, exceeding the established threshold of protective immunity.

### 3.2. HBcAg-wDIII VLPs Also Induced Antigen-Specific Cellular Immune Responses

**Figure 2:**
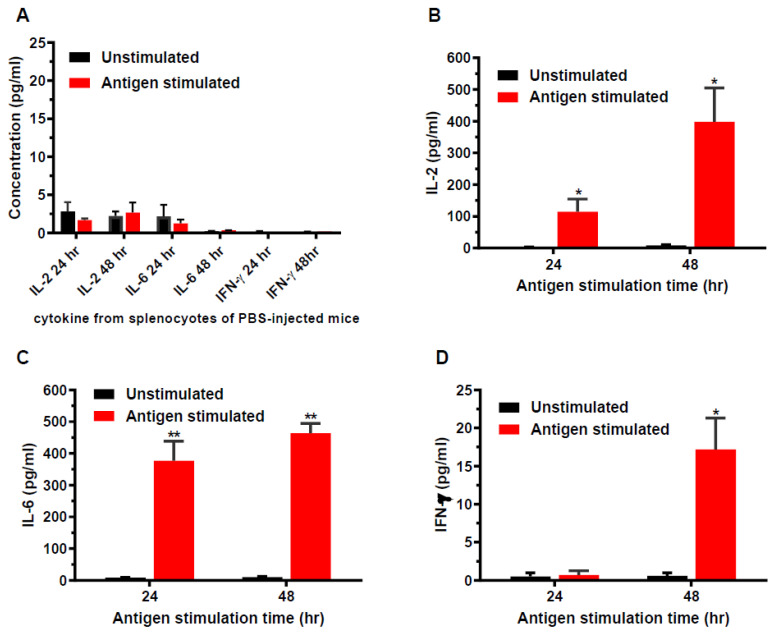
Cytokine production from splenocytes of immunized mice. Splenocytes isolated from mice mock-immunized with PBS (week 11) (**A**) or immunized with 25 µg HBcAg-wDIII VLP (**B**–**D**) were stimulated with wDIII or equivalent volume of culture media (unstimulated). After 24 or 48 h of stimulation, the levels of IL-2 (**A**,**B**), IL-6 (**A**,**C**), and IFN-γ (**A**,**D**) produced by splenocytes were quantitated. At least two independent experiments with technical triplicates were performed for each sample and the mean concentration (pg/mL) and SD are presented. Production of cytokines by antigen stimulation is statistically significant (compared to unstimulated samples, ** indicates *p* values < 0.0053 for IL-6, * indicates *p* values < 0.0174 for IL-2 24 h and 48 h, and for IFN-γ 48 h). The differences in cytokines levels between splenocytes of HBcAg-wDIII VLP-vaccinated and PBS-injected mice are also significant (*p* < 0.0112 for IL-2; *p* < 0.0120 for IL-6; and *p* < 0.0171 for IFN-γ). No significant difference in levels of cytokines was detected between unstimulated and wDIII-treated splenocytes from PBS-infected mice (*p* > 0.05).

Immunized mice were euthanized on week 11 and the spleens were aseptically removed for in vitro splenocyte cultures. The splenocytes was stimulated with wDIII antigen for 24 and 48 h, and the production of cytokines was measured to determine if HBcAg-wDIII VLPs evoked antigen-specific cellular immune responses in mice. As shown in Figure 2, splenocytes from PBS mock-immunized mice did not produce significant levels of cytokines after in vitro stimulation with wDIII (Figure 2A). In contrast, upon antigen-stimulation, significant titers of IL-2 (Figure 2B), IL-6 (Figure 2C), and IFN-γ (Figure 2D) were secreted by splenocytes from HBcAg-wDIII VLP-immunized mice. Among cytokines measured in supernatants of splenocyte cultures, the levels of IL-6 and IL-2 were significantly higher than that of IFN-γ, regardless of the duration of antigen stimulation (*p* = 0.0174 and 0.0299 for 24 h and 48 h samples of IL-6, and *p* = 0.0497 and 0.0440 for 24 h and 48 h samples of IL-2, respectively). To ensure the competency of splenocytes in producing various cytokines, ConA was used as a positive control and high levels of IL-2, IL-6, and IFN-γ were detected upon ConA stimulation.

### 3.3. HBcAg-wDIII VLPs Induced Protective Immunity against Lethal WNV Challenge

**Figure 3:**
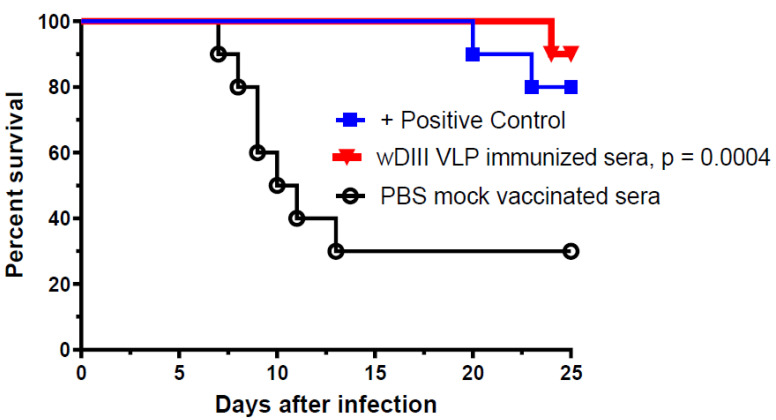
Protection of recipient mice from WNV infection by serum of HBcAg-wDIII VLP immunized mice. BALB/c mice were passively administered (r.o.) with 50 µL of heat inactivated serum collected from mice that were immunized with HBcAg-wDIII VLP (wDIII VLP) or mock-immunized with PBS. In the positive control group, 10 µg of a protective mAb was administered in place of serum. Mice were challenged with 10^2^ PFU of WNV 1 h after receiving serum or mAb. Two independent experiments with *n* = 10 mice per treatment were performed. The *p*-value (*p* = 0.0004) for HBcAg-wDIII VLP-immunized mice (compared to PBS-mock immunized) is presented.

WNV challenge studies were performed to examine if the high neutralization potency of sera from HBcAg-wDIII VLP-immunized mice would provide protective immunity against infection in recipient mice. Wild-type BALB/c mice (5-week-old, *n* = 10 per group) were first passively administered (via retro-orbital route) with heat-inactivated sera (collected at week 11) from HBcAg-wDIII VLP- or PBS-injected mice (negative control). Mice in the positive control group were injected with a protective WNV E16 mAb also via the retro-orbital route [23]. Mice were then challenged with 10^2^ PFU of WNV, which causes baseline mortality of 70–90% [23]. As expected, 70% of mice that received serum from PBS-mock immunized mice succumbed to infection and died within 13 days of WNV inoculation, while 80% of mice that received the positive control E16 mAb were protected from the viral challenge (Figure 3). Notably, serum from HBcAg-wDIII VLP-immunized mice protected 90% of recipient mice from the lethal WNV infection (*p* = 0.0004, compared to serum from PBS-mock immunized mice) (Figure 3).

### 3.4. ADE Activities of Antibodies Elicited by HBcAg-wDIII VLP Immunization

**Figure 4:**
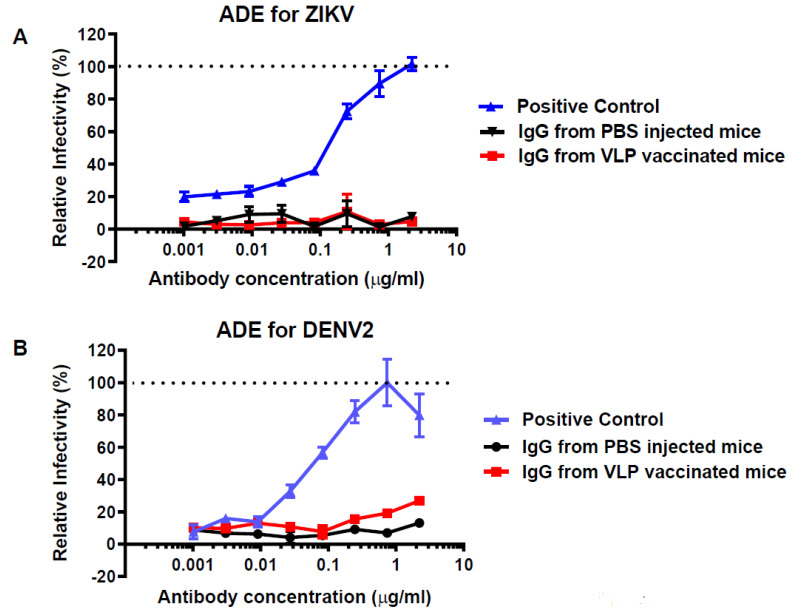
ADE activity of anti- HBcAg-wDIII VLP serum for ZIKV and DENV infection. Total serum IgG was isolated from pooled sera collected at week 11 from PBS or HBcAg-wDIII VLP- (VLP) vaccinated mice. IgGs were serially diluted, mixed with ZIKV (**A**) or DENV-2 (**B**), and incubated with FcγR-expressing K562 cells. After incubation of 72 h for ZIKV or 48 h for DENV-2, ZIKV- or DENV-2-infected cells were detected by flow cytometry. A known ADE-causing anti-flavivirus E mAb (4G2) was used as the ADE positive control (**A**,**B**). Enhancement of ZIKV or DENV infection by serum antibodies is presented as a % relative to the positive control, mAb 4G2.

Since flavivirus vaccines have the potential risk of predisposing vaccinated subjects to subsequent severe infection by related viruses via the mechanism of ADE, we investigated if antibodies evoked by HBcAg-wDIII VLP immunization would enhance ZIKV or DENV infection. As shown in Figure 4, an anti-DENV E cross-reactive mAb, 4G2 (positive control), that recognizes an epitope common to the E protein flaviviruses, effectively caused ADE of ZIKV (Figure 4A) and DENV-2 (Figure 4B) infection in K562 cells that express the human FcγR, corroborating our previous observations [20,21,22]. As expected, IgGs isolated from the negative control serum of PBS-mock immunized mice (week 11) did not promote ADE for ZIKV (Figure 4A) or DENV-2 (Figure 4B). Notably, IgGs from HBcAg-wDIII VLP-immunized mice displayed no significant ADE activity similar to IgGs from PBS-injected negative control mice for both ZIKV (Figure 4A, *p* > 0.354 for all IgG concentrations) and DENV-2 (Figure 4B, *p* > 0.177 for all IgG concentrations). In contrast, the ADE activity of IgGs from VLP-vaccinated mice is significantly different from that of the ADE-causing positive control for both ZIKV (Figure 4A, *p* < 0.0268 for all IgG concentrations) and DENV (Figure 4B, *p* < 0.034 for all concentrations except for the first three low concentrations).

## 4. Discussion

WNV has spread globally and is now endemic in many parts of the world. While the majority of WNV infections cause mild febrile illness, elderly and immune-compromised individuals are at high risk of developing lethal neuroinvasive diseases with symptoms including cognitive dysfunction and flaccid paralysis [28]. Since no licensed therapeutic is available to specifically treat WNV infection in humans, there is an urgent need for the development of vaccines to stop global outbreaks of WNV. Several types of WNV vaccine candidates are under development. They include inactivated WNV, live-chimeric virus, and DNA- or protein-based subunit vaccines with the wE protein as the main antigen [29,30,31]. Investigations of these vaccine candidates indicated that protection can be mediated by vaccine-elicited antibody response with anti-wE neutralizing titers >10 being the correlate of protective immunity [29,30,31]. While promising, these vaccine candidates still face challenges before they can be approved for human use. For example, risk factors associated with incomplete inactivation of live WNV, undesirable host responses to viral vectors, and oncogenesis due to the potential insertion of DNA vaccine fragment into the host genome all pose safety concerns. While subunit-based vaccines based on wE protein are projected to be safer compared with other candidates, their production is often impeded by low yield, limited scalability, and the need to refold the antigen and remove endotoxins [32,33,34]. The outbreaks of ZIKV in recent years have further complicated the development of WNV vaccines based on wE protein. The observation that populations with past WNV infection are more likely to develop severe symptoms during a subsequent ZIKV infection [12] has raised safety concerns for WNV vaccines in enhancing heterologous flavivirus (e.g., ZIKV and DENV) infection via ADE.

In response, we explored our plant-expression system to express wDIII, an antigen with defined neutralizing, but avoid immune-pathological epitopes in the form of VLPs, to address safety issue and vaccine production challenges since plants can produce recombinant proteins at manufacturing scale inexpensively [35,36,37] with potent efficacy [38,39,40,41]. We reported previously that high-yield production of HBcAg-wDIII VLPs was achieved in *Nicotiana benthamiana* leaves within days of introducing the target gene and the VLPs displayed the wDIII epitopes authentically on their surfaces [13]. The study reported here aims to further demonstrate the in vivo efficacy of HBcAg-wDIII VLPs and, more importantly, address ADE-related safety challenges.

We have previously demonstrated that HBcAg-wDIII VLPs not only induced the robust production of DIII-specific antibodies, but also elicited antibodies that compete with a known protective mAb in binding to the same epitope [13]. This suggests that HBcAg-wDIII VLP-evoked antibodies can be protective. Indeed, our current study confirmed such a hypothesis and demonstrated that sera collected from mice 2 weeks after the first boost of HBcAg-wDIII VLP immunization reduced WNV infection by >80% when diluted 100-fold. This indicates that HBcAg-wDIII VLP-elicited antibody response has potent neutralizing activity (neutralization titer > 100), exceeding the threshold (neutralization titer > 10) for protective immunity established by previous studies [9,22,27]. Our in vivo study further validated the protectivity of HBcAg-wDIII VLP-induced antibody immunity as anti-HBcAg-wDIII VLP sera protected 90% of recipient mice from a lethal challenge of WNV infection. These results collectively demonstrated that protective immunity against WNV infection can be achieved by a neutralizing antibody response from HBcAg-wDIII VLP immunization.

In addition to humoral responses, we also observed that wDIII-specific cellular immune responses were elicited by HBcAg-wDIII VLP immunization. Specifically, both Th1 (IFN-γ and IL-2) and Th2-type (IL-6) cytokines were induced by HBcAg-wDIII VLP administration, corroborating our previous report that wDIII can elicit both IgG1 and IgG2a responses [13]. The ability of inducing cellular immune responses by HBcAg-wDIII VLP immunization suggests its potential to clear WNV infection in addition to providing immunity against future WNV infection.

It is highly significant that our HBcAg-wDIII VLPs did not induce the production of antibodies in the immunized host that enhance the infection of DENV or ZIKV. Flavivirus vaccine development has been challenged by the risk of ADE [42]. For example, people who have antibodies from infection or vaccination for one serotype of DENV have a higher risk of developing more severe symptoms, potentially including dengue hemorrhagic fever/dengue shock syndrome (DHF/DSS), through ADE if they are infected by another serotype of DENV [10]. Studies in animal models suggest that ADE may also occur between WNV and ZIKV [12]. Therefore, minimizing ADE with heterologous flavivirus infection is one of our most important considerations for WNV vaccine development. To this end, we chose wDIII as our main antigen for our VLP-based vaccine. Overall, DIII of the E protein is less conserved than domain I and II (DI and DII) among flaviviruses [43]. As a result, antibody epitopes on DIII are generally virus-specific, and many are neutralizing and protective [44,45,46,47]. The scarce number of non-neutralizing but cross-reactive epitopes of wDIII may explain the lack of ADE activity for DENV and ZIKV infection of our HBcAg-wDIII VLP vaccine candidate. In contrast, other major WNV vaccine candidates still have the risk of inducing ADE because they contain DI and DII antigen, which may evoke cross-reactive but sub-neutralizing antibodies against ZIKV or/and DENV. A recent publication reported another plant-made VLP-based vaccine candidate that displayed wDIII on AP205 phage VLPs using the Spy-VLP technology [48]. The AP205-wDIII VLPs codelivered with Montanide Gel adjuvant are highly potent in eliciting wDIII-specific IgGs. Future comparison of neutralizing potency, cell-mediated immune response, and in vivo protection of the two wDIII-VLP-based vaccines will provide insights into how antigen presentation on VLPs affects their immunogenicity and potency. We used the passive transfer experiment to examine both the protectivity and safety of the HBcAg-wDIII VLP-induced antibodies, as this method can exclude any potential protective effects from cell-mediated immunity. Further animal experiments with a direct immunization and challenge approach and with more detailed analysis such as viremia and mouse weight loss are warranted to reveal the full protectivity of this vaccine candidate. 

In summary, we have demonstrated that a plant-produced VLP vaccine candidate can protect mice against WNV infection. More importantly, this vaccine candidate may have a safety advantage over others due to the lack of ADE activity for ZIKV and DENV infection. Thus, our study may promote the development of WNV vaccines that are potent, safe, and can be produced at a large scale with low cost.

## Data Availability

The data presented in this study are contained within this article.

## Supplementary Materials

The following supporting information can be downloaded at: https://www.mdpi.com/article/10.3390/vaccines11020197/s1, Figure S1. Neutralization of WNV by mouse serum collected 11 week after HBcAg-wDIII VLP immunization.
