# Supplementary material for: Antibody-Dependent Enhancement Activity of a Plant-Made Vaccine against West Nile Virus
Source: Vaccines (Basel). 2023 Jan 17;11(2):197. doi: 10.3390/vaccines11020197 (PMC9966755; doi:10.3390/vaccines11020197)
Supplement: Supplementary file 1 [file vaccines-11-00197-s001.zip › vaccines-2128473-supplementary.pdf]

Supplementary material

Material and methods

Plaque reduction neutralization test (PRNT) assay

WNV (CT2741) was diluted to a working concentration of 10^2 PFU per well in Opti-MEM serum free medium (ThermoFisher, NY). Serum samples were diluted 1:10 and 1:100 in the same medium. WNV was then incubated with diluted sera for 1 hr at 37°C, followed by transferring the virus/serum mixture to plates containing 90-95% confluent monolayers of Vero cells (ATCC # CCL-81). After 1 hr of incubation at 37°C, virus/serum-containing medium were removed and Vero cells were overlaid with fresh MEM medium containing 1% SeaPlaque™ agarose (Lonza, MD). Cell monolayer was stained with 4% (vol/vol) neutral red after an additional 72 hr of incubation and plaques were counted. Percent (%) neutralization was calculated as: [(number of WNV plaque per well without test serum) - (number of WNV plaque per well of diluted test serum) / (number of WNV plaque per well without test serum) x 100].

Cytokine production in splenocytes

Mechanical dissociation method was employed to establish splenocyte cultures (5×10^6 cells /ml) from spleens isolated from immunized mice. Splenocytes were stimulated with 10 μg/ml of wDIII. For positive and negative controls, T cell mitogen Con A (positive control, 5 μg/ml, MilliporeSigma, MA) and culture medium (negative control) were used in place of wDIII antigen, respectively. After 24 and 48 hr of stimulation, the supernatant of spleen cell cultures was collected and quantitated for IL-2, IL-6, and IFN-γ levels by using a custom mouse cytokine kit (Bio-Rad, CA) following the manufacturer’s protocol. The Bio-Plex 200 system and the associated Bio-Plex Manager software (version 5.0, Bio-Rad, CA) were used for data collection and analysis from at least two independent experiments with triplicates for each cytokine.

Antibody-dependent enhancement Assay

Total IgG was isolated from pooled sera collected at week 11 from vaccinated mice using IgG purification kits (GE Healthcare, PA). Serial dilutions of serum-derived IgGs were incubated with DENV-2 (ATCC#VR-1584) or ZIKV (PRVABC59, ATCC# VR-1843) at 37°C for 1 hr and then added to FcγRIIA$^+$ K562 cells (ATCC # CCL-2243) at an MOI of 1. After 48 hr (DENV-2) or 72 hr (ZIKV) of incubation at 37°C, K562 cells were collected, fixed with 4% paraformaldehyde (MilliporeSigma, MA), and permeabilized with 0.1% saponin (MilliporeSigma, MA). Infected K562 cells were then stained with anti-flavivirus antibody that is fluorescently labeled with Alexa Fluor488 (Invitrogen, CA) and detected by flow cytometry. An ADE-causing anti-flavivirus E mAb (4G2) (ATCC # HB-112) was used as an ADE positive control.

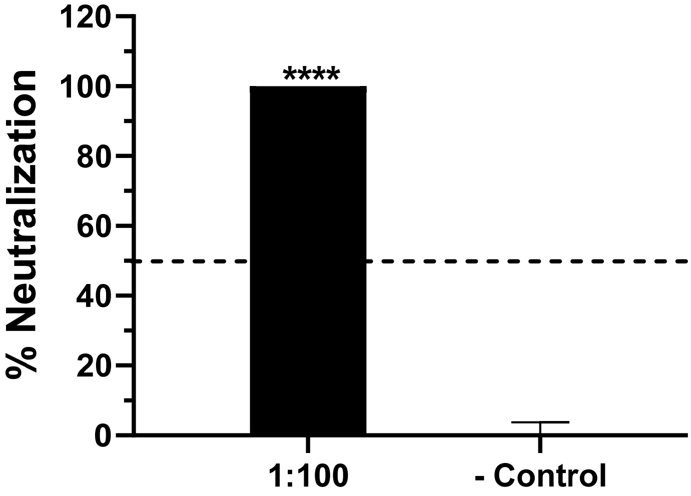

Figure S1. Neutralization of WNV by mouse serum collected 11 week after HBcAg-wDIII VLP immunization. The neutralization potency against WNV of pooled sera from mice that were immunized with HBcAg-wDIII VLP was measured by a PRNT assay. Sera were diluted 100 folds and incubated with 10^2 PFU of WNV prior to infection of Vero cells. Mean % neutralization two independent experiments with technical triplicates for each sample are presented.

– Control: PBS buffer in the PRNT assay. **** indicates p values < 0.0001 of HBcAg-wDIII VLP-immunized sera compared to that from PBS-injected control mice.
